# Co-infection of Sweet Orange with Severe and Mild Strains of *Citrus tristeza virus* Is Overwhelmingly Dominated by the Severe Strain on Both the Transcriptional and Biological Levels

**DOI:** 10.3389/fpls.2017.01419

**Published:** 2017-08-31

**Authors:** Shimin Fu, Jonathan Shao, Changyong Zhou, John S. Hartung

**Affiliations:** ^1^Citrus Research Institute, Southwest University Chongqing, China; ^2^Molecular Plant Pathology Laboratory, United States Department of Agriculture-Agricultural Research Service Beltsville, MD, United States

**Keywords:** *Citrus sinensis*, transcriptome, host–pathogen interaction, defense response, closterovirus

## Abstract

Citrus tristeza is one of the most destructive citrus diseases and is caused by the phloem-restricted Closterovirus, *Citrus tristeza virus*. Mild strain CTV-B2 does not cause obvious symptoms on indicators whereas severe strain CTV-B6 causes symptoms, including stem pitting, cupping, yellowing, and stiffening of leaves, and vein corking. Our laboratory has previously characterized changes in transcription in sweet orange separately infected with CTV-B2 and CTV-B6. In the present study, transcriptome analysis of *Citrus sinensis* in response to double infection by CTV-B2 and CTV-B6 was carried out. Four hundred and eleven transcripts were up-regulated and 356 transcripts were down-regulated prior to the onset of symptoms. Repressed genes were overwhelmingly associated with photosynthesis, and carbon and nucleic acid metabolism. Expression of genes related to the glycolytic, oxidative pentose phosphate (OPP), tricarboxylic acid cycle (TCA) pathways, tetrapyrrole synthesis, redox homeostasis, nucleotide metabolism, protein synthesis and post translational protein modification and folding, and cell organization were all reduced. Ribosomal composition was also greatly altered in response to infection by CTV-B2/CTV-B6. Genes that were induced were related to cell wall structure, secondary and hormone metabolism, responses to biotic stress, regulation of transcription, signaling, and secondary metabolism. Transport systems dedicated to metal ions were especially disturbed and ZIPs (Zinc Transporter Precursors) showed different expression patterns in response to co-infection by CTV-B2/CTV-B6 and single infection by CTV-B2. Host plants experienced root decline that may have contributed to Zn, Fe, and other nutrient deficiencies. Though defense responses, such as, strengthening of the cell wall, alteration of hormone metabolism, secondary metabolites, and signaling pathways, were activated, these defense responses did not suppress the spread of the pathogens and the development of symptoms. The mild strain CTV-B2 did not provide a useful level of cross-protection to citrus against the severe strain CTV-B6.

## Introduction

Citrus tristeza has historically been one of the most destructive and globally distributed citrus diseases and is still responsible for tremendous economic losses to the citrus industries worldwide (Bennett and Costa, 1949; Moreno et al., 2008). The disease is caused by *Citrus tristeza virus* (CTV), a member of the *Closteroviridae*, with single-stranded and positive-sense genomic RNA (gRNA) of about ~19 kb in size (Karasev et al., 1995; Moreno et al., 2008). CTV is phloem-restricted and long-distance spread of the virus is via movement of infected plants or propagation of infected buds. It is also transmitted locally by several aphid species in a semi-persistent mode, most notably by *Toxoptera citricida* (Kirkaldy). Strains of CTV can be classified as mild to severe based on the type and intensity of symptoms caused in different citrus hosts. Mild CTV strains do not cause symptoms on most indicators and usually do not bring about economic loss, while severe CTV strains induce distinct disease syndromes, including quick decline and death of sweet orange on sour orange rootstocks, or stem pitting (SP) of sweet orange and grapefruit scions when grown on other rootstocks, seedling yellows (SY) in sour orange, and vein clearing in “Mexican” lime seedlings (Dawson et al., 2013). Based on phylogenetic analysis of nucleotide sequences of the most variable genomic region, CTV has been divided into seven genotypes: T3, T30, T36, VT, B165/T68, RB, and HA16-5 (Dawson et al., 2015). It is interesting that complete symptoms of *Tristeza* disease were observed in transgenic Mexican lime plants that expressed the product of CTV ORF 11, an RNA binding protein designated p23 (Ghorbel et al., 2001).

Physiological and cellular changes caused by virus infection are accompanied by differential gene expression in plants. Understanding host responses to infection by pathogens facilitates the understanding of plant–pathogen interactions and also provides insights for new control strategies. Changes in the transcriptomes of susceptible Mexican lime (Gandía et al., 2007; Liu et al., 2012; Yang et al., 2013) and sweet orange (Cheng et al., 2016) and resistant trifoliate orange (Cristofani-Yaly et al., 2007) in response to the infection by mild or severe CTV strains have been well documented. Changes in gene expression in resistant, tolerant, and susceptible hosts in response to CTV were also compared to huanglongbing (HLB; Bowman and Albrecht, 2015). Citrus hosts are commonly infected with more than one CTV strain, sometimes as many as seven strains in a single tree (Roy et al., 2010). Some mild CTV strains have played an important role in protecting susceptible commercial varieties in citrus industries worldwide by cross protection (Grant and Costa, 1951), but failure is common (Roistacher and Dodds, 1993) because the presumptive protecting strain must be selected to match the genotype of the strains of CTV in the local environment (Folimonova and Achor, 2010).

Sweet orange (*Citrus sinensis*) is susceptible to CTV and accounts for ~60% of citrus production (Moreno et al., 2008; Xu et al., 2013). In a previous study, transcriptional changes in sweet orange separately infected with CTV-B2 and CTV-B6 revealed a disturbance of circadian rhythm and ionic homeostasis, as well as activation of defense responses including modification of cell walls. The regulation of transcription, hormone, and secondary metabolism, are also affected by infection by CTV, but with differing patterns depending on the strain of CTV (Fu et al., 2016). In this study, transcriptome data were collected from sweet orange co-infected with both mild strain CTV-B2 and severe strain CTV-B6. This provides new insight into the host response to simultaneous infection with mild and severe strains of CTV and how these changes are correlated with the host response to infection by single strains of CTV.

## Materials and methods

### Plant materials and inoculation with CTV

CTV mild strain B2 (T30 genotype, Florida) and severe strain B6 (mixed genotype, SY568, California; Vives et al., 2005; Ruiz-Ruiz et al., 2006) are maintained *in planta* as part of the Exotic Pathogens of Citrus Collection (EPCC) at the USDA-ARS Beltsville Agricultural Research Center (BARC) in Beltsville, MD. Both CTV-B2 and CTV-B6 were graft-inoculated into ten “Valencia” sweet orange seedlings simultaneously. Ten trees were mock-inoculated with their own buds as the healthy control.

### Extraction of RNA and detection of CTV

Inoculated plants were tested for the presence of CTV specific amplicons by RT-PCR as described (Roy et al., 2010). RNA extracts were prepared for RNA-Seq immediately after the trees became RT-PCR positive and before any symptoms developed from three young, soft, not fully expanded leaves of uniform size. Leaves were frozen in liquid nitrogen and kept at −80°C until the extraction of RNA and assessment for quantity and quality with Qubit (Thermo-Fisher, Pittsburgh, PA) and Bioanalyzer (Agilent, Santa Clara, CA) instruments. Three qualified RNA replicates, each of three leaves from different plants, for CTV-B2/CTV-B6 and healthy citrus were sent to Otogenetics (Norcross, GA, USA) for paired-end sequencing with the Illumina HiSeq 2500 platform.

### Statistical analyses and RT-qPCR verification

Raw reads obtained by Illumina HiSeq 2500 were filtered to exclude low complexity reads. Clean reads from the nine libraries were aligned to the reference genome (Xu et al., 2013) with Bowtie (Langmead, 2010) and fold changes (log_2_ FC) for each transcript were evaluated by DEseq2 (Love et al., 2014). Differentially expressed transcripts (DETs) were filtered with cut-off values, Padj ≤ 0.1, |log_2_ FC| ≥ 1 and e-value ≤ e^−5^. DETs involved in pathways were enriched with Panther (Mi et al., 2009) and the Kyoto Encyclopedia of Genes and Genomes (KEGG) data bases. A summary of gene expression patterns was visualized with MapMan software (Thimm et al., 2004). Twenty genes were selected and transcripts were amplified by RT-qPCR to validate the RNASeq analysis as described (Fu et al., 2016). Primers used for RT-qPCR were designed by Real-time PCR online tool (Integrated DNA Technology, IDT, Table S1).

In order to estimate the relative concentration of CTV-B2 and CTV-B6 genomes in co-infected plants, RNA-Seq libraries from trees co-inoculated with CTV-B2 and CTV-B6 and mock inoculated trees were searched for sequences homologous to the p23 protein of reference CTV T30 genome (AF260651) and the reference CTV T318A genome (DQ151548). The 100 bp paired end reads were mapped to the reference genomes under stringent conditions, allowing a maximum of 1 mismatch per read duo to the high homology between the p23 protein of CTV-B2 and CTV-B6.

## Results

### Infection and symptoms

**Figure 1:**
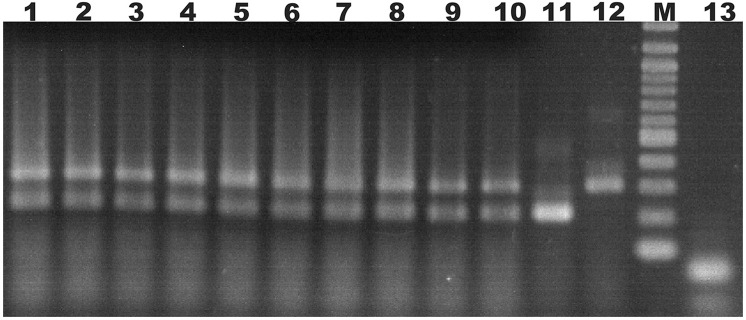
Agarose gel electrophoresis of products of RT-PCR of RNA extracts from sweet orange seedlings to confirm co-infection. Lanes 1–10, extracts of trees simultaneously infected with *Citrus tristeza virus* strains CTV-B2 and CTV-B6; 11 and 12, sweet orange seedlings inoculated with strain CTV-B2 and CTV-B6 only; M, 100 base pair ladder; 13, extracts from healthy sweet orange seedlings. Amplicons of 206 and 302 bp are specific for CTV-B2 and CTV-B6, respectively.

**Figure 2:**
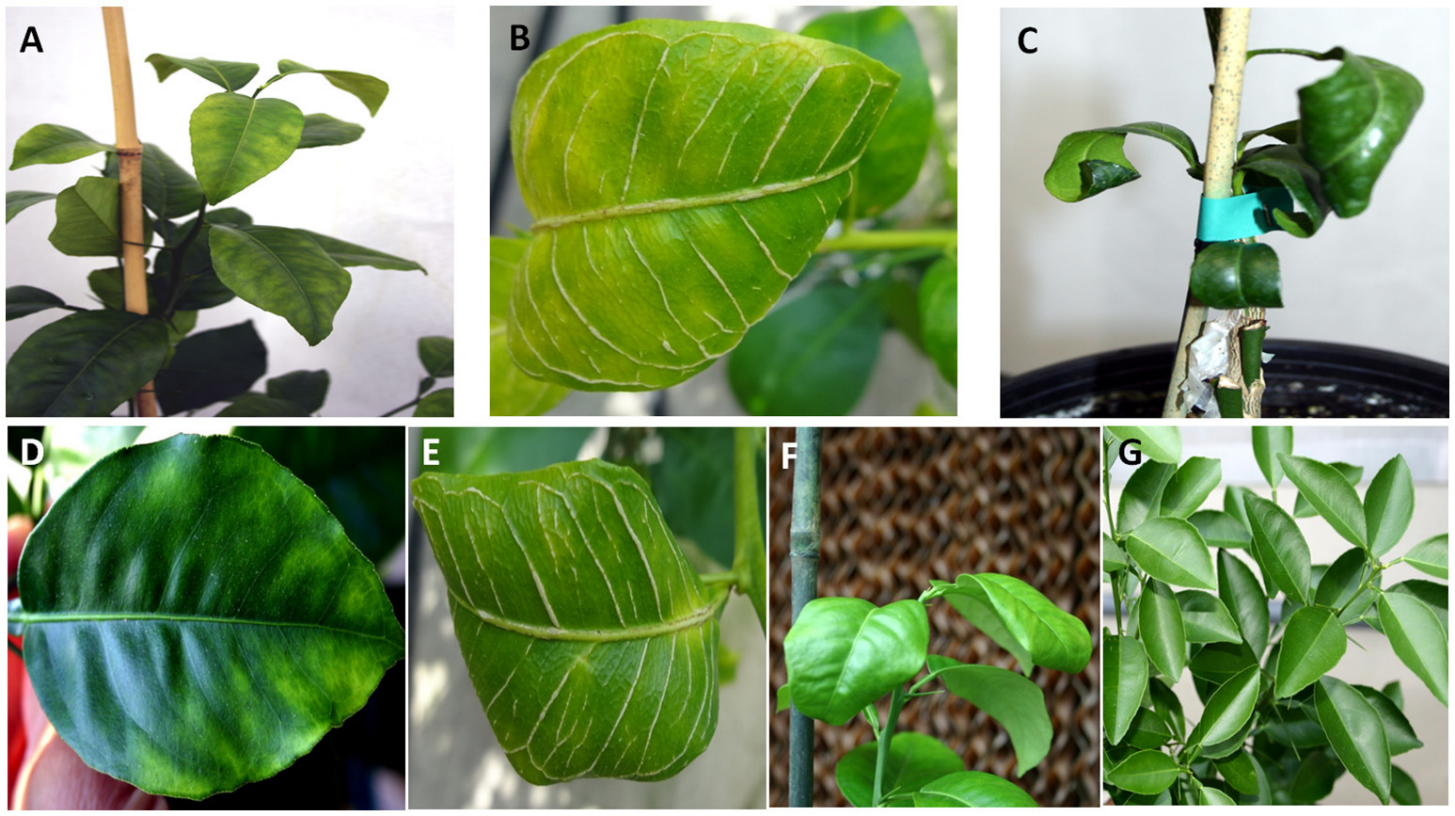
Leaf symptoms in sweet orange seedlings infected with *Citrus tristeza virus* strains CTV-B2 and CTV-B6 together and separately. **(A–C)** Seedlings co-infected with CTV-B2 and CTV-B6, **(D–F)** seedlings infected with CTV-B6 alone, **(G)** seedlings infected with CTV-B2 alone, **(A,D)** chlorosis; **(B,E)** vein corking; **(C,F)** leaf curl; **(G)** no symptoms.

Ten doubly-inoculated trees became positive for both mild CTV-B2 and severe CTV-B6 based on RT-PCR after 2–4 months (Figure 1). Symptoms developed 5–6 months after inoculation. Trees infected with CTV-B2/CTV-B6 were much smaller and stunted than the healthy controls, with leaf chlorosis, vein corking, and vein curling (Figure 2) increasing as time passed. Symptoms of the co-inoculated plants 12 months after co-inoculation were indistinguishable from those produced by plants infected with CTV-B6 alone. In the plants co-inoculated with CTV-B2 and CTV-B6, the p23 transcript of CTV-B6 was found 300 times more frequently than the CTV-B2 homolog. Sequences homologous to p23 were not found in the mock-inoculated controls, nor when other libraries that contained only CTV-B2 or CTV-B6 were searched for the heterologous p23 sequence.

### Overview of RNA-seq

**Figure 3:**
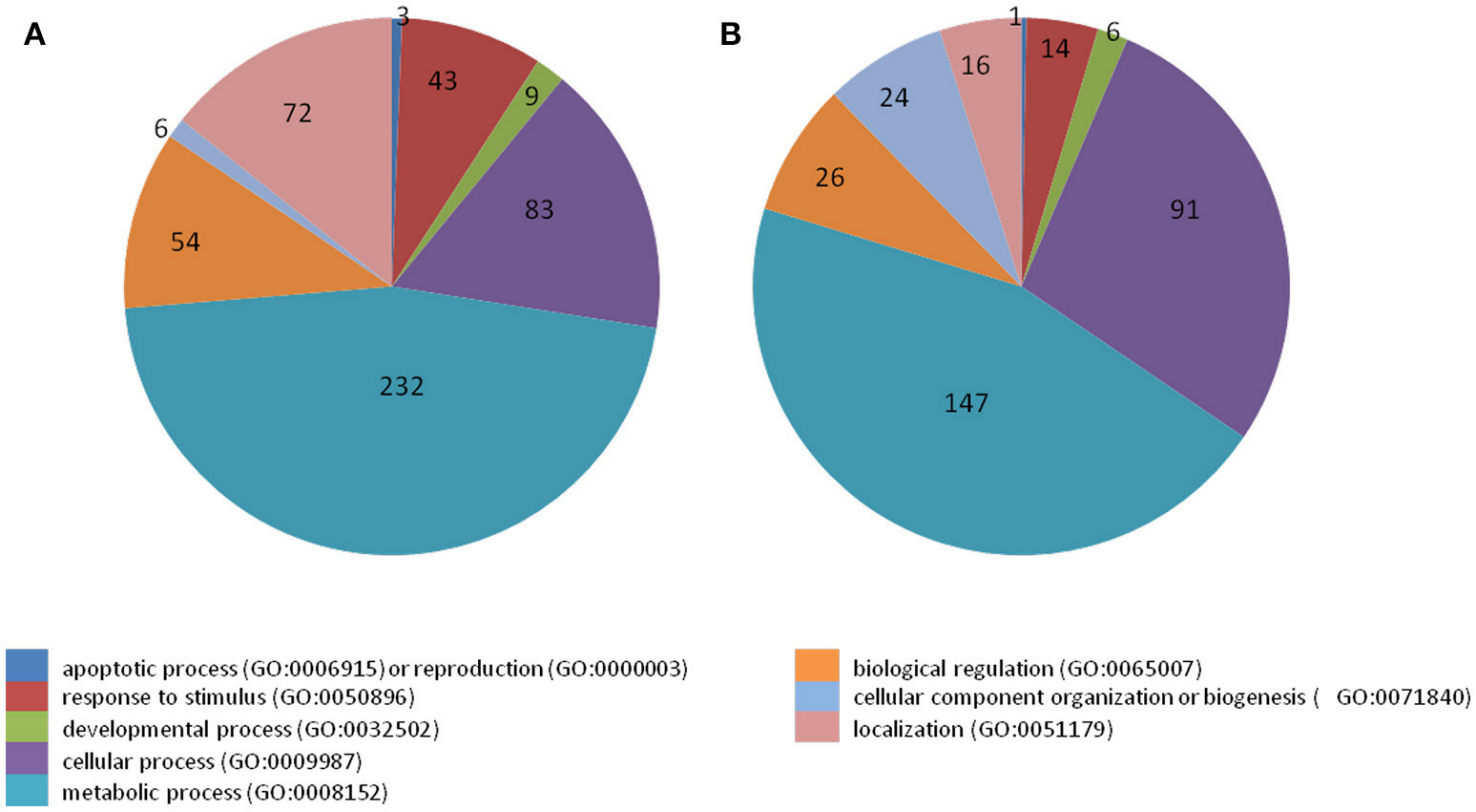
Categorization of transcripts induced or repressed in *Citrus sinensis* co-infected with *Citrus tristeza virus* strains CTV-B2 and CTV-B6 compared with the healthy control. **(A)** Induced transcripts; **(B)** repressed transcripts.

**Figure 4:**
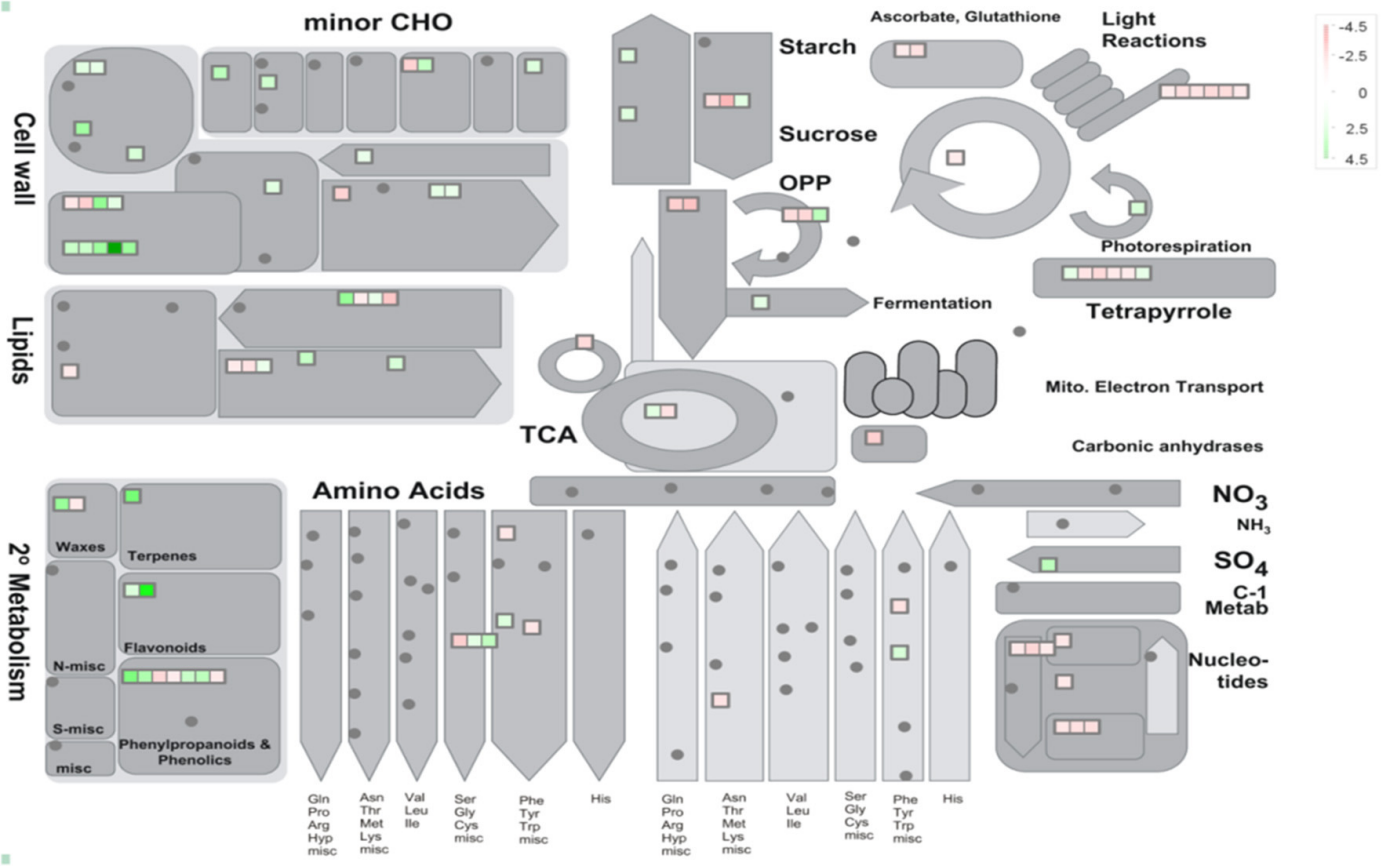
Metabolic overview of young asymptomatic sweet orange leaves in response to co-infection by *Citrus tristeza virus* strains CTV-B2 and CTV-B6. Green and red squares are shaded to represent the degree of up- and down-regulation of transcripts in response to CTV-B2 and CTV-B6 co-infection, respectively. Gray circles represent transcripts without differential expression between infected and healthy plants.

**Figure 5:**
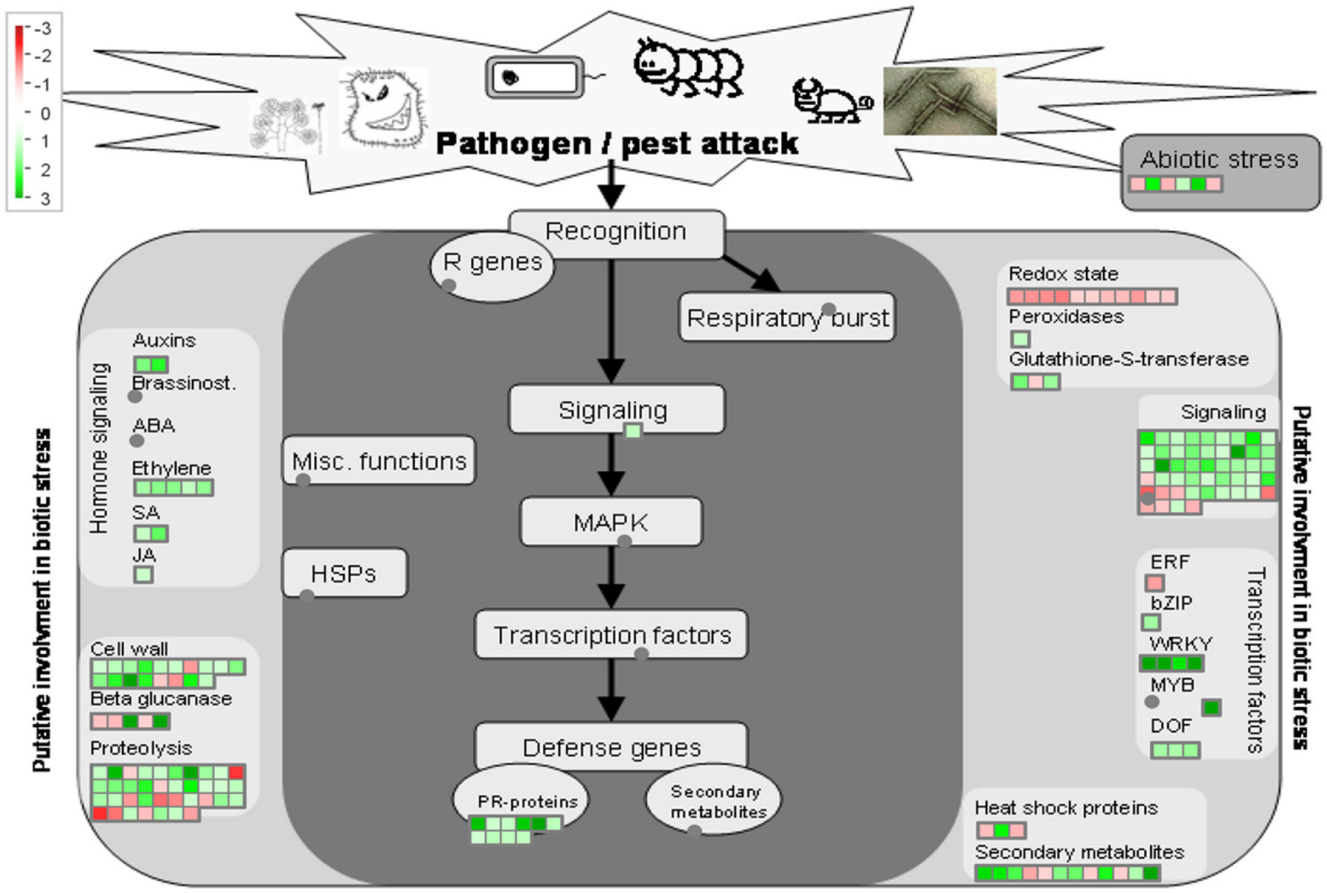
Overview of biotic stress pathways in young asymptomatic sweet orange leaves in response to co-infection by *Citrus tristeza virus* strains CTV-B2 and CTV-B6. Green and red squares are shaded to represent the degree of up- and down-regulation of transcripts in response to CTV-B2 and CTV-B6 co-infection, respectively. Gray circles represent transcripts in pathways without change between infected and healthy plants.

38 to 47 million raw reads were obtained from each inoculated tree with approximate 61% (average of three replicates) of these reads successfully aligned to the *C. sinensis* reference genome (Xu et al., 2013; Figure S1). Compared with mock-inoculated healthy trees, a total of 767 transcripts were differentially expressed: 411 were induced and 356 were repressed (Padj ≤ 0.1; Figure S2). DETs were found to be enriched for different biological processes (level 2) with Panther based on orthologs of *Arabidopsis thaliana*. The distribution of functional categories for up- and down-regulated genes was similar: metabolic processes, cellular processes, localization, biological regulation, and response to stimulus (Figure 3). The set of down-regulated genes had relatively more genes related to cellular component organization/biogenesis but less for localization (Figure 3). In an overview of metabolic and biotic stress pathways, genes related to light reactions, oxidative pentose phosphate (OPP), tricarboxylic acid cycle (TCA), tetrapyrrole synthesis, nucleotide metabolism, carbohydrate, and lipid metabolism were mostly repressed (Figure 4), whereas the genes associated with cell wall synthesis, secondary and hormone metabolism, pathogenesis-related (PR) proteins, and signaling were mostly induced (Figure 5). A full list of differentially expressed transcripts is shown in more detail (Table S2).

**Figure 6:**
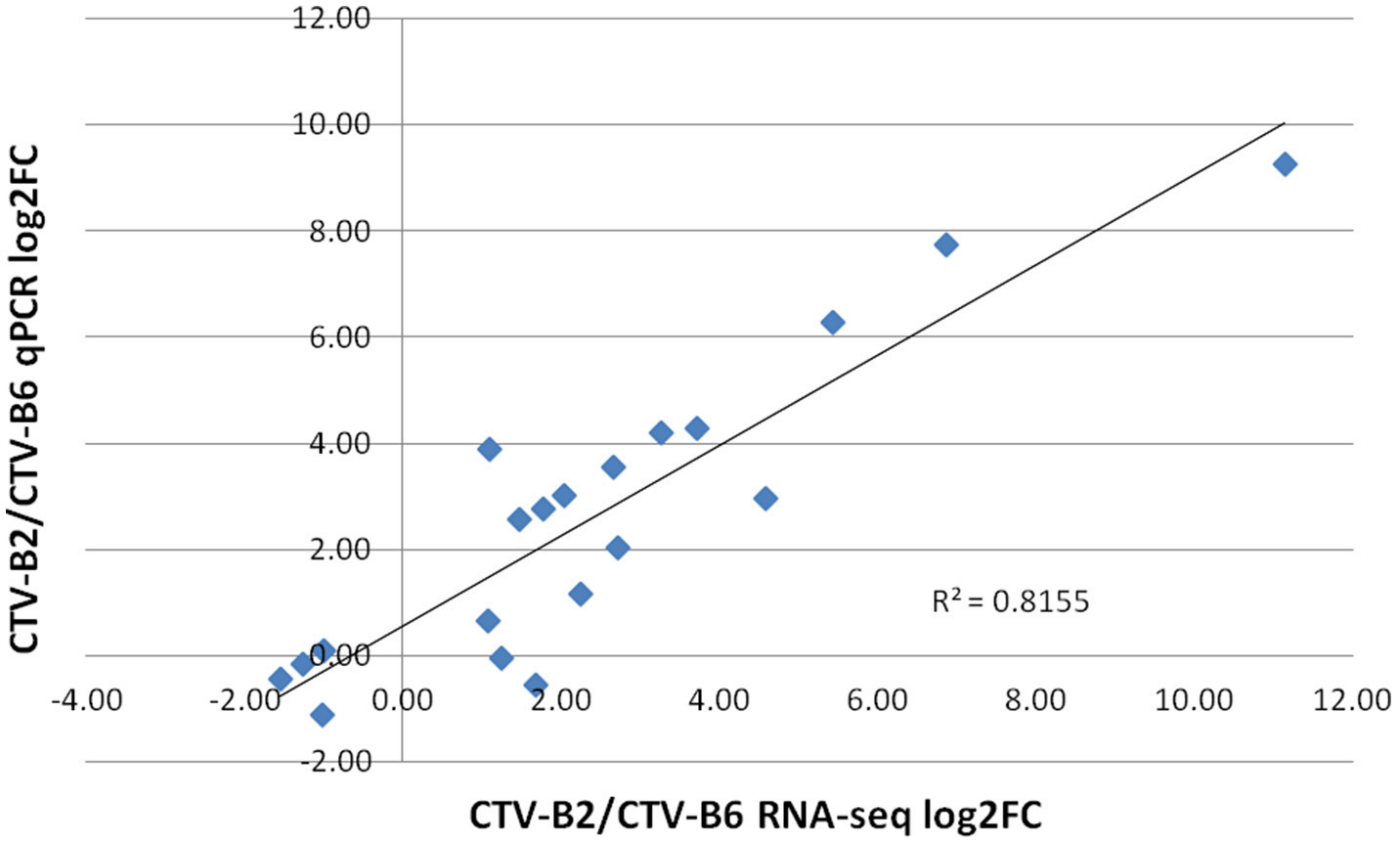
Correlation of estimates of fold change of differentially expressed transcripts by RT-qPCR and RNA-seq. log_2_FC, Fold Change.

The expression levels of selected DETs ranged from −2 to 8 (log_2_ FC). Similar expression patterns were obtained for all evaluated transcripts by both RNA-seq and RT-qPCR techniques. The dissociation pattern and single peak obtained after RT-qPCR verified the specificity of RT-qPCR primers (Figure S3). A high Spearman's rho value (0.82) indicated good correlation of gene expression between the RNA-seq and RT-qPCR results and confirmed the reliability and accuracy of the RNA-seq data in our study (Figure 6).

### Photosynthesis, nucleic acid, and iron metabolism

The expression of several photosynthesis-related genes was down-regulated in response to CTV-B2/CTV-B6. These genes included *LHCA4* (light-harvesting chlorophyll-protein complex I subunit A4), *PPL1* (PsbP-like protein 1), thylakoid lumenal 20 kDa protein, *PSAD-1* (photosystem I subunit D-1), photosystem II reaction center *PsbP* family protein, and *PTAC14* (plastid transcriptionally active14; Table 1). Genes in the tetrapyrrole synthesis pathway were also repressed, including *GSA2* (glutamate-1-semialdehyde 2, 1-aminomutase 2), *HEMC* (hydroxymethylbilane synthase), *HEME2* (uroporphyrinogen decarboxylase), and *HEMF1* (coproporphyrinogen oxidase; Table 1).

**Table 1:** Down-regulated transcripts in *Citrus sinensis* in response to infection by CTV-B2/CTV-B6.

**Gene symbol**	**Transcript id_ *Citrus sinensis***	**AGI**	**Gene description**	**Fold change**
**PS AND TETRAPYRROLE SYNTHESIS**				
LHCA4	Orange1.1g025674m	AT3G47470	Light-harvesting chlorophyll-protein complex I subunit A4	−1.01
PPL1	Orange1.1g025977m	AT3G55330	Psbp-like protein 1	−1.27
TLP	Orange1.1g023424m	AT3G56650	Thylakoid lumenal 20 Kda Protein	−1.19
PSAD-1	Orange1.1g047658m	AT4G02770	Photosystem I subunit D-1	−1.32
PTAC14	Orange1.1g011739m	AT4G20130	Plastid transcriptionally active14	−1.00
GSA2	Orange1.1g011959m	AT3G48730	Glutamate-1-Semialdehyde 2,1-Aminomutase 2	−1.19
HEMC	Orange1.1g020472m	AT5G08280	Hydroxymethylbilane synthase	−1.38
HEME2	Orange1.1g016596m	AT2G40490	Uroporphyrinogen decarboxylase	−1.06
HEMF1	Orange1.1g016102m	AT1G03475	Lesion initiation 2	−1.03
ATM3	Orange1.1g030870m	AT2G15570	Thioredoxin M-Type 3, Chloroplast (Trx-M3)	−1.51
ATM3	Orange1.1g030784m	AT2G15570	Thioredoxin M-Type 3, Chloroplast (Trx-M3)	−1.58
ACHT5	Orange1.1g023089m	AT5G61440	Atypical Cys His Rich Thioredoxin 5	−1.71
ACHT4	Orange1.1g022923m	AT1G08570	Atypical Cys His Rich Thioredoxin 4	−1.02
FSD3	Orange1.1g025006m	AT5G23310	Fe superoxide dismutase 3	−1.07
TAPX	Orange1.1g016584m	AT1G77490	Thylakoidal ascorbate peroxidase	−1.02
APX6	Orange1.1g019824m	AT4G32320	L-Ascorbate peroxidase/Heme binding/Peroxidase	−1.23
**NUCLEOTIDE METABOLISM**				
PYR4	Orange1.1g020186m	AT4G22930	Pyrimidin 4	−1.43
NDPK2	Orange1.1g026906m	AT5G63310	Nucleoside diphosphate kinase 2	−1.06
RNR1	Orange1.1g003561m	AT2G21790	Ribonucleotide reductase 1	−1.24
MCM3	Orange1.1g004502m	AT5G46280	DNA replication licensing factor, putative	−1.04
MCM2	Orange1.1g002353m	AT1G44900	ATP binding/DNA binding	−1.27
MCM5	Orange1.1g004862m	AT2G07690	Minichromosome maintenance family protein	−1.43
MCM7	Orange1.1g005024m	AT4G02060	Prolifera	−1.45
FAS1	Orange1.1g003501m	AT1G65470	Fasciata 1	−1.15
RPA70B	Orange1.1g006973m	AT5G08020	Rpa70-Kda subunit B	−1.64
HIS H4	Orange1.1g047769m	AT5G59970	Histone H4	−2.14
HIS H3.2	Orange1.1g032664m	AT4G40030	Histone H3.2	−2.10
HTA7	Orange1.1g032326m	AT5G27670	Histone H2A 7; DNA binding	−2.27

Genes encoding enzymes for nucleotide metabolism *PYR4* (pyrimidine 4), *NDPK2* (nucleoside diphosphate kinase 2), and *RNR1* (ribonucleotide reductase 1) were repressed. Genes related to DNA synthesis were also down-regulated, as were some genes that encode DNA repair proteins *MCM2* (DNA replication licensing factor), *MCM3, FAS1* (fasciata 1), *RPA70B* (rpa70-kda subunit B), *PRL* (prolifera), and genes for several histones, including *H3, H4*, and *HTA7* (Table 1).

### Alteration of ribosomal composition

A large group of genes encoding ribosomal proteins were repressed, including components of the 30S, 40S, 50S, and 60S subunits, indicating an extensive reprogramming of ribosomal synthesis (Table 2). Ribosomal proteins (RPs) play roles in metabolism, growth and cell division, but also function in developmental processes as regulatory components (Byrne, 2009).

**Table 2:** Transcripts encoding ribosomal proteins were overwhelmingly down-regulated in *Citrus sinensis* in response to infection by CTV-B2/CTV-B6.

**Gene symbol**	**Transcript id_*Citrus sinensis***	**AGI**	**Gene description**	**Fold change**
RPS5	Orange1.1g021869m	AT2G33800	Ribosomal protein S5 family protein	−1.11
RPS6	Orange1.1g027743m	AT1G64510	Ribosomal protein S6 family protein	−1.26
RPS9	Orange1.1g042358m	AT1G74970	Ribosomal protein S9; structural constituent of ribosome	−1.13
RPS10	Orange1.1g041275m	AT3G13120	30S ribosomal protein S10, chloroplast, putative	−1.35
RPS17	Orange1.1g033970m	AT1G79850	Ribosomal protein S17; structural constituent of ribosome	−1.01
RPL3	Orange1.1g023905m	AT2G43030	Ribosomal protein L3 family protein	−1.17
RPL5	Orange1.1g024440m	AT4G01310	Ribosomal protein L5 family protein	−1.21
RPL9	Orange1.1g029153m	AT3G44890	Ribosomal protein L9; structural constituent of ribosome	−1.16
RPL13	Orange1.1g026067m	AT1G78630	Embryo defective 1473; structural constituent of ribosome	−1.07
RPL13	Orange1.1g028533m	AT1G78630	Embryo defective 1473; structural constituent of ribosome	−1.09
RPL15	Orange1.1g024515m	AT3G25920	Structural constituent of ribosome	−1.05
RPL17	Orange1.1g041707m	AT3G54210	Ribosomal protein L17 family protein	−1.30
RPL18	Orange1.1g030804m	AT1G48350	Ribosomal protein L18 family protein	−1.33
RPL21	Orange1.1g027519m	AT1G35680	50S Ribosomal protein L21, chloroplast/CL21 (RPL21)	−1.12
RPL24	Orange1.1g029417m	AT5G54600	50S Ribosomal protein L24, chloroplast (CL24)	−1.34
RPL34	Orange1.1g031306m	AT1G29070	Ribosomal protein L34 family protein	−1.33
RPS27	Orange1.1g034124m	AT5G44710	Molecular_function unknown	1.01
RPS21	Orange1.1g030080m	AT3G27160	Structural constituent of ribosome	−1.23
RPL35	Orange1.1g031865m	AT2G24090	Ribosomal protein L35 family protein	−1.18
RPS4B	Orange1.1g024793m	AT5G07090	40S Ribosomal protein S4 (RPS4B)	−1.05
RPS20B	Orange1.1g033482m	AT3G47370	40S Ribosomal protein S20 (RPS20B)	−1.18
RPS24B	Orange1.1g032727m	AT5G28060	40S Ribosomal protein S24 (RPS24B)	−1.47
RPS25	Orange1.1g033931m	AT4G34555	40S Ribosomal protein S25, putative	−1.01
ARP1	Orange1.1g038172m	AT1G43170	Arabidopsis Ribosomal Protein 1	−1.21
RPL15A	Orange1.1g028730m	AT4G16720	60S Ribosomal protein L15 (RPL15A)	−1.11
RPL37C	Orange1.1g034400m	AT3G16080	60S Ribosomal protein L37 (RPL37C)	−1.03
^*^	Orange1.1g034489m	AT5G40080	60S Ribosomal protein-related	1.27
RPL18AA	Orange1.1g035537m	AT1G29970	60S Ribosomal protein L18A-1	−1.24
RPL37A	Orange1.1g044880m	AT3G10950	60S Ribosomal protein L37a (RPL37aB)	−1.04
^*^	Orange1.1g026076m	AT5G38290	Ribosomal biogenesis, peptidyl-tRNA hydrolase	−1.00
^*^	Orange1.1g043340m	AT2G39670	Radical SAM domain-containing protein	−1.63

* *Transcripts without gene symbol*.

### Cell wall barriers were enhanced

The expression of a majority of genes for cell wall metabolism was up-regulated: Genes encoding *CESA9* (cellulose synthase A9) and *PRP4* (cell wall proline-rich protein 4), as well as *XTH28* (xyloglucan endotransglycosylase) and *XTH30* were all up-regulated and the expression level of *EXLB3* (expansin-like B3 precursor) was up-regulated ~7 fold (Table 3).

**Table 3:** Up-regulated transcripts in *Citrus sinensis* in response to infection by CTV-B2/CTV-B6.

**Gene symbol**	**Transcript id_ *Citrus sinensis***	**AGI**	**Gene description**	**Fold change**
**CELL WALL**				
CESA9	Orange1.1g001373m	AT2G21770	Cellulose synthase A9	1.25
PRP4	Orange1.1g029350m	AT4G38770	Proline-rich protein 4	1.43
XTR2, XTH28	Orange1.1g019909m	AT1G14720	Xyloglucan endotransglycosylase related 2	1.66
XTR4, XTH30	Orange1.1g018153m	AT1G32170	Xyloglucan endotransglycosylase 4	2.24
EXLB1	Orange1.1g025808m	AT4G17030	*Arabidopsis thaliana* expansin-like B1	2.21
EXLB3	Orange1.1g032956m	AT2G18660	Expansin-like B3 Precursor	6.86
**METAL HANDLING AND TRANSPORT**				
FRO6	Orange1.1g006160m	AT5G49730	Ferric reduction oxidase 6	1.38
FP6	Orange1.1g031705m	AT4G38580	Farnesylated protein 6; metal ion binding	1.71
ZIP1	Orange1.1g018051m	AT3G12750	Zinc transporter 1 precursor	11.13
ZIP5	Orange1.1g044821m	AT1G05300	Metal ion transmembrane transporter	5.44
ZIP11	Orange1.1g044590m	AT1G55910	Zinc transporter 11 precursor	1.30
AAP6	Orange1.1g011548m	AT5G49630	Amino acid permease 6	1.30
AAP7	Orange1.1g012732m	AT5G23810	Amino acid transmembrane transporter	1.34
AST56, SULTR2;2	Orange1.1g008914m	AT1G77990	Sulfate transmembrane transporter	1.29
AST68, SULTR2;1	Orange1.1g006023m	AT5G10180	Sulfate transmembrane transporter	1.07
NRT1.1	Orange1.1g007736m	AT1G12110	Nitrate transmembrane transporter	1.38
ABC	Orange1.1g045930m	AT1G51460	ABC transporter family protein	2.95
**DEFENSE RELATED**				
TPS21	Orange1.1g043754m	AT5G23960	Terpene synthase 21	2.65
CER3	Orange1.1g006767m	AT5G57800	Eceriferum 3	2.28
TT4	Orange1.1g016330m	AT5G13930	Transparent testa 4	1.20
AFB5	Orange1.1g044749m	AT5G49980	Auxin F-box protein 5	1.72
GA5	Orange1.1g016776m	AT4G25420	GA5	1.64
ERS1	Orange1.1g005591m	AT2G40940	Ethylene response sensor 1	1.63
EIN4	Orange1.1g004510m	AT3G04580	Ethylene insensitive 4	1.20
RLP21	Orange1.1g021196m	AT2G25470	Receptor like protein 21	1.40
CHIB1	Orange1.1g044801m	AT5G24090	Acidic endochitinase	3.13
EDS1	Orange1.1g007278m	AT3G48090	Enhanced disease susceptibility 1	1.20
NBS-LRR class	Orange1.1g046345m	AT4G27190	Disease resistance protein	1.17
NBS-LRR class	Orange1.1g040659m	AT5G63020	Disease resistance protein	1.08
RLP1	Orange1.1g042603m	AT1G07390	Protein binding	2.03
RLP6	Orange1.1g038037m	AT1G45616	Receptor like protein 6	2.43
RLP15	Orange1.1g001036m	AT1G74190	Receptor like protein 15	4.48
CES101	Orange1.1g004402m	AT3G16030	Callus expression of Rbcs 101	1.58
IQD23	Orange1.1g013059m	AT5G62070	IQ-domain 23	2.18
FRS5	Orange1.1g002062m	AT4G38180	Far1-related sequence 5	1.10

### Transportation system

Transcription of genes related to transport and metal binding were largely up-regulated. These included transporters for nucleotides, sugars, amino acids, sulfate, phosphate, peptides, and oligopeptides and ABC transporters, such as, *AAP6* and *AAP7* (amino acid trans membrane transporter), *AST56* and *AST68* (sulfate transporter), *NRT1.1* (nitrate transporter), *ZIP1, ZIP5*, and *ZIP11* (zinc ion trans membrane transporter; Table 3). Transcripts encoding phloem protein PP2-B13 were somewhat down-regulated in response to co-infection with CTV-B2/CTV-B6, but were strongly down-regulated by CTV-B2 alone, whereas transcripts for PP2-B15 were slightly up-regulated by single infection with CTV-B6 (Fu et al., 2016).

### Up-regulation of hormone and secondary metabolism and signaling associated defense responses

Genes involved in the synthesis of secondary metabolites and hormone metabolism were induced, such as, *TPS21* (terpene synthase 21), *CER3* (ceriferum 3), *TT4* (transparent testa 4), *AFB5* (auxin F-Box protein 5), *GA5* (gibberellin 20-oxidase), *EIN4* (ethylene insensitive 4), and *ERS1* (ethylene response sensor 1). Genes for biotic stress associated proteins *RLP21* (Receptor Like Protein 21), *CHIB1* (acidic endochitinase) and *EDS1* (enhanced disease susceptibility 1), and disease resistance proteins in the CC-NBS-LRR class were also induced (Table 3). Genes for receptor kinases, calcium and light related signaling were mostly induced, such as, *RLP1* (receptor like protein 1), *RLP6*, and *RLP15, CSE101* (callus expression of rbcs 101), *IQD23* (IQ-domain 23) binding calmodulin, and *FRS5* (FAR1-related sequence 5) binding zinc ions (Table 3).

## Discussion

### Infection and symptoms

Both strains of CTV were able to establish infections in the inoculated sweet orange plants, indicating that simultaneous inoculation of CTV-B2 and CTV-B6 did not prevent infection and establishment of either strain in sweet orange. We isolated RNA from the inoculated plants after infection was established but before symptoms were expressed. The assessment of gene expression prior to the onset of visible symptoms allowed identification of changes in gene expression that led to the symptoms that were subsequently observed. Symptoms of infection by strain CTV-B2 are very mild and difficult to observe. However, in our doubly inoculated plants the symptoms were extensive and severe. CTV-B6 has a complex genome based on components of several genotypes (Ruiz-Ruiz et al., 2006), and the nucleotide sequence of the p23 gene is 98.5% identical to the reference strains T318A. In our co-inoculated plant, the p23 transcript of CTV-B6 was found much more frequently than the CTV-B2 homolog, consistent with the symptoms induced in the plants (Ghorbel et al., 2001). In cases where strains are simultaneously present in sweet orange, the genotype of the severe component often becomes dominant (Sambade et al., 2007). This is evidence that CTV-B6 is the dominant strain when trees are co-infected with mild CTV-B2 and the severe, virulent CTV-B6, and that CTV-B2 is not able to protect sweet orange from CTV-B6.

### Photosynthesis, nucleic acid, and iron metabolism

Genes that encode proteins that are components of both the light harvesting complex and that contribute to the assembly of tetrapyrroles and chlorophyll were down regulated. In addition to photosynthesis, tetrapyrroles play crucial roles in a broad range of biological processes including respiration and assimilation of nitrogen/sulfur in higher plants (Tanaka et al., 2011). In our study, genes associated with photosynthesis and tetrapyrrole metabolism were mostly down-regulated in response to CTV-B2/CTV-B6. HEMC, HEME2, and HEMF1 are enzyme intermediates in tetrapyrrole synthesis and their down-regulation indicates a disturbance of tetrapyrrole synthesis, as well as chlorophyll synthesis in host plants, because chlorophyll biosynthesis has been suggested to depend upon a balance between the methyl erythritol phosphate and tetrapyrrole pathways (Kim et al., 2013). The PsbP protein is indispensible for the regulation/stabilization (Ifuku et al., 2005) and photo autotrophy (Yi et al., 2007) of the PSII complex, as well as normal thylakoid membrane architecture (Yi et al., 2009) in higher plants. PsbP-like proteins (PPLs) are PsbP homologs and PPL1 is required for efficient repair of photo damaged PSII in higher plants (Ishihara et al., 2007). These alterations in gene expression patterns are consistent with altered thylakoid membrane structure and function in response to CTV infection. The decrease of several photosynthetic proteins (i.e., PSAD, PsbP, and LHCA) would also cause defects in photosynthetic performance (Romani et al., 2012). Symptoms such as, yellowing and chlorosis of leaves caused by CTV-B6 or CTV-B2/CTV-B6 may be due in part to the decrease of the PsbP andPPLs, as observed in experiments with PsbP-deficient tobacco (*Nicotiana tabacum*) which had pale-green-colored leaves (Yi et al., 2009). The decrease in the levels of these proteins limits plant growth as there is less carbohydrate synthesized as a result of the repressed photosynthesis.

Iron metabolism was also dramatically altered in response to infection by CTV-B2 and CTV-B6, which likely contributed to the alterations photosynthetic processes observed. As we observed with infection of citrus with CTV-B2/CTV-B6, genes related to photosynthesis and tetrapyrrole metabolism were also dramatically repressed in *Arabidopsis* leaves in response to iron (Fe) deficiency (Rodríguez-Celma et al., 2013). Iron has crucial effects on respiration and tetrapyrrole metabolism and its deficiency compromises chlorophyll synthesis, causing chlorosis in developing leaves and decreased photosynthetic activity. The iron deficiency may be exacerbated by the decline of feeder roots induced by CTV-B6, which limit the uptake of iron from the rhizosphere.

The overwhelming down-regulation of genes related to DNA synthesis is also in concert with iron deficiency as iron is required in many enzymatic reactions in the DNA replication pathway. Hexameric mini chromosome maintenance proteins (MCMs) have important roles in DNA replication in plants (Ni et al., 2009; Herridge et al., 2014) and some of them are critical for DNA unwinding. *MCM2* modulates root meristem function in *Arabidopsis* (Ni et al., 2009). The down regulation of *MCM2* is consistent with the decline in feeder roots seen as the CTV syndrome develops. *MCM2* was also down-regulated in rice with limited nutrients (Cho et al., 2008). In *A. thaliana*, MCM3 is present in various oligomeric forms including as a homohexamer and possesses 3′ to 5′ helicase and ATPase activities *in vitro* (Rizvi et al., 2015). MCM7 protein is localized in the nucleus and is required for DNA replication and cytokinesis at an early stage of *Arabidopsis* development (Springer et al., 2000; Holding and Springer, 2002). The repression of MCM2, MCM3, MCM5, and MCM7 was associated with viral infection previously (Choi et al., 2015), but detailed functional studies have not been carried out. The down-regulation of photosynthetic capability, tetrapyrrole and lipid metabolism and DNA replication are all associated with iron deficiency. Similar symptoms of leaf yellowing following disturbances in heavy metal metabolism are found in citrus suffering from huanglongbing (HLB). In the case of HLB, foliar sprays of nutritional supplements alleviate symptoms (Spann and Schumann, 2009; Stansly et al., 2013), and may be worthwhile in the case of severe CTV as well.

### Alteration of ribosomal composition

Reduction of shoot growth and cell proliferation were found in ribosomal protein mutants (Byrne, 2009; Horiguchi et al., 2011, 2012). Many chloroplast ribosomal protein genes were strongly down-regulated in a related study in response to co-infection with CTV-B6 and “*Candidatus* Liberibacter asiaticus,” causal agent of citrus huanglongbing (Bové, 2006; submitted), and were also greatly repressed in response to CTV-B2/CTV-B6, including *RPS17, RPL9, RPL17*, and *RPL18* (Table 2). Gene expression in chloroplasts is primarily controlled through the regulation of translation, mediated by the ribosome. Great alterations of ribosomal composition were observed in leaves and roots of *Arabidopsis* in response to iron and phosphate deficiency (Rodríguez-Celma et al., 2013; Wang et al., 2013). Gene expression in chloroplasts was limited and further affected the photosynthesis capacity.

### Cell wall barriers were enhanced

Cellulose is the most abundant and strongest component of cell walls and it is targeted for degradation by pathogens to facilitate penetration of and movement between plant cells. Genes associated with cellulose synthase were induced by double infection with CTV-B2/CTV-B6 and by single infection with CTV-B2 but not by CTV-B6 alone (Fu et al., 2016). *XTH* gene expression and enzyme activity was positively associated with cell wall elongation (Shin et al., 2006) and strengthening (Antosiewicz et al., 1997). Expansin activity is most often in association with cell-wall loosening in growing cells (Lee et al., 2001), and expansin proteins are distributed throughout cell walls (Zhang and Hasenstein, 2000; Cosgrove et al., 2002). Both *XTH* and *EXLB* are also correlated with cell wall modification, which is an important aspect of plant responses to biotic and abiotic stresses, and is likely to be the relevant context in this study. The expression of *EXLB3* was also up-regulated by single infection with CTV-B2 (Table 4), but not as strongly as in response to double infection with CTV-B2/CTV-B6. The stronger up-regulation of *EXLB3* may be stimulated by the presence of virulent CTV-B6. The up-regulation of *XTH* and *EXLB* gene expression when under virus attack can modulate cell wall growth and equip the plant with a stronger physical barrier to deter feeding by the vector aphids. The remodeling and thickening of cell walls is also correlated with the symptoms of stiff and thickened leaves and vein corking (Figure 2).

**Table 4:** Transcripts in *Citrus sinensis* with shared differential expression pattern when co-infected with both CTV-B2/CTV-B6 and with each virus alone.

**Gene symbol**	**Transcript id_**	**AGI**	**Gene description**	**Fold change**		
	***Citrus sinensis***			**CTV-B2/CTV-B6**	**CTV-B2^a^**	**CTV-B6^a^**
^*^	Orange1.1g021031m	AT4G38690	1-phosphatidylinositol phosphodiesterase-related	1.70		1.10
^*^	Orange1.1g012242m	AT3G29635	Transferase family protein	1.35		1.30
DMR6	Orange1.1g019665m	AT5G24530	DOWNY MILDEW RESISTANT 6	1.42	1.90	2.80
RLP21	Orange1.1g021196m	AT2G25470	Receptor like protein 21	2.56		1.40
^*^	Orange1.1g029641m	AT1G58170	Disease resistance-responsive protein-related	5.97		1.80
^*^	Orange1.1g028960m	AT2G20560	DNAJ heat shock family protein	2.43		1.80
TT4	Orange1.1g016330m	AT5G13930	Transparent testa 4	3.44		1.20
TT7	Orange1.1g010388m	AT5G07990	Transparent testa 7	1.36	2.90	2.90
RDR1	Orange1.1g003789m	AT1G14790	RNA-dependent RNA polymerase1	2.40		1.70
^*^	Orange1.1g021502m	AT1G28310	Dof-type zinc finger domain-containing protein	1.45		1.30
WRKY70	Orange1.1g020291m	AT3G56400	Transcription factor/transcription repressor	3.72	1.80	3.90
WRKY50	Orange1.1g029257m	AT5G26170	Transcription factor	2.59	2.10	
WRKY60	Orange1.1g032690m	N	Rice TFs having WRKY and zinc finger domains	3.93		3.20
WRKY60	Orange1.1g033112m	N	Rice TFs having WRKY and zinc finger domains	3.84		3.90
^*^	Orange1.1g040562m	AT1G64830	Aspartyl protease family protein	8.23		3.10
^*^	Orange1.1g011556m	AT5G10770	Chloroplast nucleoid DNA-binding protein, putative	3.46		2.00
CDR1	Orange1.1g014537m	AT5G33340	CONSTITUTIVE DISEASE RESISTANCE 1	5.39		3.90
^*^	Orange1.1g002533m	AT5G39000	Protein kinase family protein	1.07		1.30
FER	Orange1.1g048439m	AT3G51550	FER (FERONIA); kinase/protein kinase	3.17		1.20
BCS1	Orange1.1g024550m	AT3G50930	Cytochrome BC1 synthesis	1.76		1.40
^*^	Orange1.1g021013m	AT5G25560	Zinc finger (C3HC4-type RING finger) family protein	1.22		1.60
EBF1	Orange1.1g006749m	AT2G25490	EIN3-binding F box protein1	1.52		−1.20
ARK3	Orange1.1g006915m	AT4G21380	*A. thaliana* receptor kinase 3	1.35		1.20
PLA2A	Orange1.1g015047m	AT2G26560	Phospholipase A 2A	2.93		1.70
ZIP11	Orange1.1g044590m	AT1G55910	ZINC transporter 11 precusor	2.04	1.10	1.30
ZIP1	Orange1.1g018051m	AT3G12750	ZINC transporter 1 precursor	11.13	−2.50	
ZIP5	Orange1.1g044821m	AT1G05300	Cation transmembrane transporter	5.44	2.40	
BAT1	Orange1.1g010352m	AT2G01170	Bidirectional amino acid transporter 1	1.23	1.10	
ATPUP5	Orange1.1g017963m	AT2G24220	Purine transmembrane transporter	1.90	1.10	
^*^	Orange1.1g031629m	AT1G14870	INVOLVED IN: response to oxidative stress	2.87		1.30
^*^	Orange1.1g048792m	AT3G02650	Pentatrico peptide (PPR) repeat-containing protein	3.01		2.70
^*^	Orange1.1g021539m	AT2G23520	Catalytic/pyridoxal phosphate binding	1.40		−1.10
^*^	Orange1.1g039041m	AT3G48770	ATP binding/DNA binding	2.78		1.90
^*^	Orange1.1g000623m	AT3G48770	ATP binding/DNA binding	2.63		1.90
^*^	Orange1.1g014290m	AT4G31980	Unknown protein	2.74		1.50
^*^	Orange1.1g022937m	AT3G12320	Unknown protein	−1.19		−1.90
^*^	Orange1.1g000988m	AT3G48770	ATP binding/DNA binding	1.43		1.20
PCK1	Orange1.1g005865m	AT4G37870	Phosphoenolpyruvate carboxykinase 1	−1.36	1.10	
EXLB3	Orange1.1g032956m	AT2G18660	Expansin-like B3 precursor	6.86	1.20	
^*^	Orange1.1g003996m	AT4G27190	Disease resistance protein (NBS-LRR class), putative	1.41	1.40	
^*^	Orange1.1g040489m	AT2G12190	Cytochrome P450, putative	1.57	1.30	

* *Transcripts without gene symbol*.

*N, Transcripts without corresponding AGI number*.

a *Data in columns “CTV-B2” and “CTV-B6” cited from Fu et al. (2016)*.

### Transportation system

Induction of transporters was also observed in a previous study (Fu et al., 2016). The up-regulation of sugar, amino acid, sulfate, phosphate, and metal and oligopeptide transporters denoted a shortage of these substances and a disturbance of the transportation system in young leaves prior to the development of symptoms. *SUC2* (sucrose-proton symporter 2), encoding a sucrose/proton symporter was up-regulated and is necessary for phloem loading and is also fundamental for phloem transport and plant productivity (Srivastava et al., 2009).

Ammonium is the preferred form of nitrogen for uptake by plants (Gazzarrini et al., 1999) as it requires less energy for assimilation into amino acids (Bloom et al., 1992). The assimilation of ammonium from the soil solution is regulated by ammonium transporters (AMT) and *AtAMT1;1* is expressed in roots and leaves (Engineer and Kranz, 2007).

The differential down-regulation level of phloem proteins in response to co-infection with CTV-B2/CTV-B6 and single infection with CTV-B2 are consistent with the restructuring of phloem, as well as with cell-to-cell and long distance movement of viruses (Imlau et al., 1999; Gómez and Pallás, 2004).

The up-regulation of *ZIP11* was also observed in response to single infections with CTV-B2 or CTV-B6 (Table 4; Fu et al., 2016). However, the expression of *ZIP5*, as well as *ZIP1, ZIP4*, and was down-regulated by CTV-B2 in a single infection (Table 4). Zinc is a key element possessing both structural and functional roles in plants and about 4% of all predicted proteins in *Arabidopsis* contain one or more zinc fingers (Kawagashira et al., 2001). A large number of enzymes contain zinc binding sites, such as, copper-zinc superoxide dismutase (Hacisalihoglu et al., 2003) and zinc metallo proteases (Ståhl et al., 2002). The differential expression patterns of *ZIP1, ZIP4*, and *ZIP5* in response to co-infection by CTV-B2/CTV-B6 and infection by CTV-B2 alone is probably due to severe, virulent CTV-B6, which affects the plant profoundly, inducing chlorosis of leaves, collapse of phloem and root decline.

Iron is also an essential metal nutrient required for a number of critical cellular functions such as, the synthesis of heme and heme-dependent oxygen transport, iron-dependent enzymatic reactions, photosynthesis and ribonucleotide synthesis (Straus, 1994; Kobayashi and Nishizawa, 2012). *FRO6* (ferric reduction oxidase 6) was up-regulated in response to CTV-B2/CTV-B6 infection. *FRO6* is involved in metal binding/acquisition and is responsible for regulating the reduction of iron from ferric to ferrous at the plasma membrane of leaf cells in response to iron deficiency (Maynes, 2013). Compared to roots of iron-sufficient plants, very high concentrations of manganese, zinc and copper accumulated in roots of iron-deficient *Arabidopsis* plants (Korshunova et al., 1999; Connolly et al., 2002; Vert et al., 2002). Hence, the high up-regulation of zinc transporters may be a secondary effect of iron deficiency.

### Up-regulation of hormone and secondary metabolism and signaling associated defense responses

Auxin regulates gene expression and facilitates elongation of shoot cells by activating the auxin response factor (ARF) family of transcription factors. Auxin is perceived by TIR1/AFB (transport inhibitor response1/auxin signaling F-BOX) family of F-box proteins (Salehin et al., 2015). In the presence of auxin, members of TIR1/AFB family of proteins regulate the polyubiquitylation and proteasomal degradation of Aux/IAA transcriptional repressors (Dharmasiri et al., 2005; Greenham et al., 2011). ARFs are repressed by the Aux/IAA proteins. Some of the most common symptoms produced by plant viruses are leaf curling, chlorosis, and stunting, are related to disruptions of plant hormone production, accumulation, and sensing (Jameson and Clarke, 2002; Padmanabhan et al., 2005). The auxin system has been directly disrupted by viral components in other systems. Auxin signaling was disrupted by the *Tobacco mosaic virus* (TMV) 126 KDa replication protein via an interaction with select Aux/IAA family members (Padmanabhan et al., 2005, 2006), which are removed by auxin-induced degradation. The up regulation of *AFB5* and down regulation of *ARF18* and *ARF19* are signs of alterations in auxin metabolism, which may be associated with disease symptoms such as, stunting and leaf curling.

Ethylene is a vital growth regulator and is also important for biotic and abiotic stress responses (Schaller, 2012). Ethylene is perceived by a family of five membrane-associated receptors *(ETR1, ETR2, ERS1, ERS2*, and *EIN4)*. The ethylene receptors are involved in transcriptional as well as post-transcriptional regulatory mechanisms (O'Malley et al., 2005; Chen et al., 2007; Kevany et al., 2007). The ethylene receptors function as negative regulators of ethylene responses and their function is inactivated through ethylene binding (Bleecker and Kende, 2000; Chang and Stadler, 2001). The up regulation of *ERS1* and *EIN4* imply enhanced ethylene production, which is an early and active response of plants to virus attack in association with the induction of defense responses (Boller, 1991).

Gibberellins are master regulators for plant growth in response to both abiotic (temperature, salt, and light) and biotic stress (Claeys et al., 2014; Colebrook et al., 2014). A giberellin-responsive pathway regulates photosynthesis (Xie et al., 2016). The up-regulation of *AFB5*, auxin-responsive protein, *ERS1* and *EIN4*, and gibberellin-responsive protein are changes of hormone signaling pathways in plants under pathogen stress and lead to the induction of the following defense actions.

Terpenoids are a structurally diverse group of plant secondary metabolites and are involved in both direct and indirect plant defenses. Terpene synthases (TPSs) play an important role in the synthesis of volatile terpenes. The expression of *TPS21* can be induced by gibberellin and jasmonate and the induction of *TPS21* increases the emission of sesquiterpenes. The plant cuticle plays a vital role in the interactions between the plant and environment stresses, including water stress. Alkanes are prominent components of cuticular wax and the alkane-forming pathway is controlled by the co-expression of *CER1/CER3* (eceriferum; Bernard et al., 2012). The increased expression of *CER1/CER3* increases the amount of wax on the surface of the leaves. It is probable that increasing expression of these genes in association with hormone and signaling pathways mediated by secondary metabolites enhanced the tolerance of the plants to water stress and may deter feeding by the aphid vector of CTV.

When mild CTV-B2 and severe CTV-B6 were inoculated simultaneously, both mild and severe CTV strains successfully established in citrus plants. The sweet orange plants co-infected with both CTV strains showed symptoms typical of CTV-B6, indicating virulent CTV-B6 is the dominant strain and that neither virus affected the other's establishment in sweet orange. The failure of CTV-B2 to cross protect is likely because the two virus strains are from different genotypes (Folimonova et al., 2010; Folimonova, 2013). The photosynthetic and carbon metabolism capacities of host plants were greatly reduced, and the composition of the ribosome was greatly altered in response to co-infection. These responses may be attributed to deficiency of iron, and secondarily to zinc deficiency. Host plant metal ion transport systems were generally disturbed and ZIPs showed very different expression patterns in response to co-infection by CTV-B2/CTV-B6 and to single strain infection by CTV-B2, which was due to the presence of virulent CTV-B6.

The outcome of a plant–virus interaction from antagonism to mutualism is determined by the environment, host genotype, and pathogens. Virulence is the negative impact of parasite infection on host fitness (Read, 1994; Little et al., 2010) and is determined by both the host and pathogen. Host defenses may decrease virulence through either resistance or tolerance. The former limits the multiplication of the parasite and the latter decreases damage to the host in spite of the amount of multiplication by the pathogen. *C. sinensis* is generally susceptible to CTV. Though defense responses, such as, strengthening of cell walls, altered hormone metabolism, increased production of secondary metabolites and signaling proteins were induced, these did not suppress the spread of the pathogens and the development of symptoms caused by CTV-B2/CTV-B6, or strain CTV-B6 alone, although they were able to induce tolerance to strain CTV-B2 (Fu et al., 2016).

## Author contributions

SF: performed the laboratory experiments and drafted the manuscript; SF and JS: collected data and carried out all data analyses; JH: designed the study, oversaw the research and co-wrote the manuscript; CZ: revised the manuscript.

### Conflict of interest statement

The authors declare that the research was conducted in the absence of any commercial or financial relationships that could be construed as a potential conflict of interest.
