# Supplementary material for: Co-infection of Sweet Orange with Severe and Mild Strains of Citrus tristeza virus Is Overwhelmingly Dominated by the Severe Strain on Both the Transcriptional and Biological Levels
Source: Front Plant Sci. 2017 Aug 31;8:1419. doi: 10.3389/fpls.2017.01419 (PMC5583216; doi:10.3389/fpls.2017.01419)
Supplement: Supplementary file 3 [file Image1.PDF]

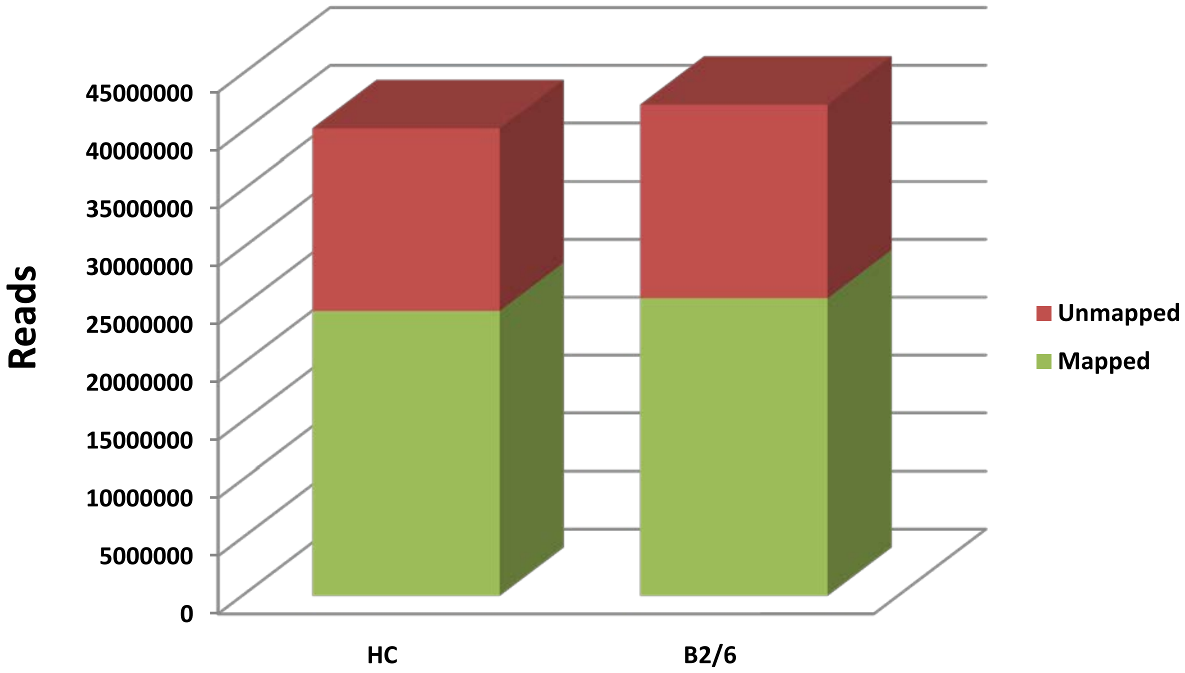

Figure S1 Summary of reads from healthy *Citrus sinensis* and *C. sinensis* infected with citrus tristeza virus strains CTV-B2 and CTV-B6. Reads were obtained through pair-end RNA sequencing and mapped to the *C. sinensis* genome
