# Supplementary material for: Co-infection of Sweet Orange with Severe and Mild Strains of Citrus tristeza virus Is Overwhelmingly Dominated by the Severe Strain on Both the Transcriptional and Biological Levels
Source: Front Plant Sci. 2017 Aug 31;8:1419. doi: 10.3389/fpls.2017.01419 (PMC5583216; doi:10.3389/fpls.2017.01419)
Supplement: Supplementary file 4 [file Image2.PDF]

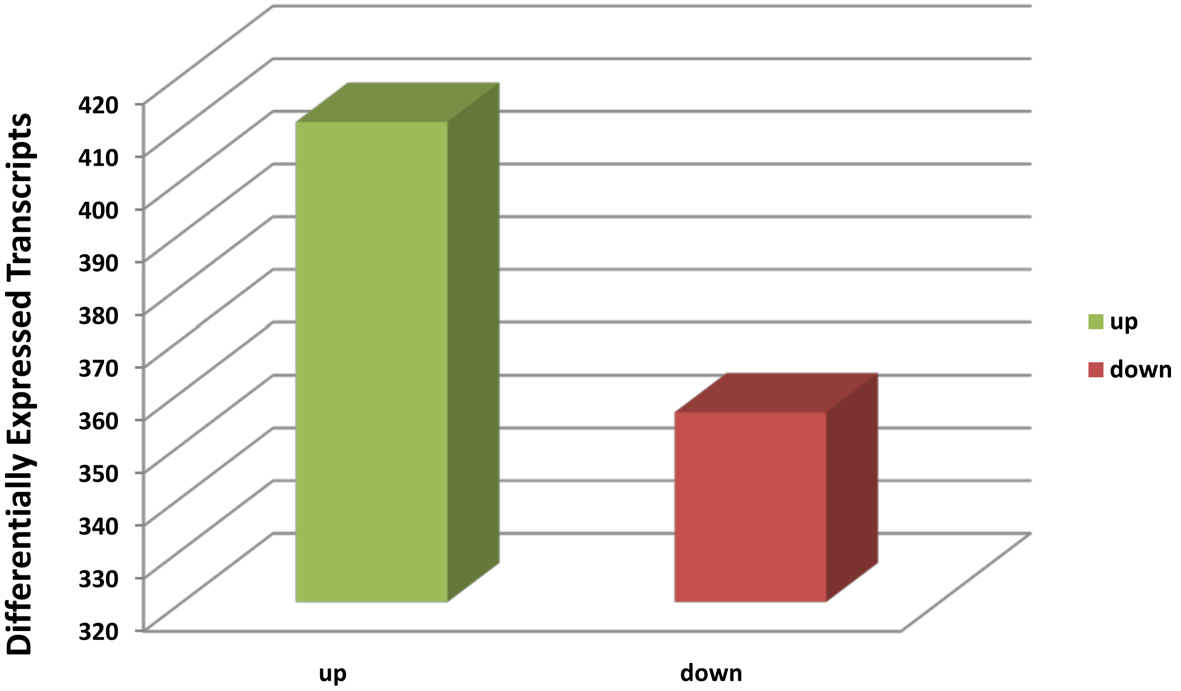

Figure S2 Transcripts differentially expressed in *Citrus sinensis* in response to co-infection by citrus tristeza virus strains CTV-B2 and CTV-B6 compared to healthy *C. sinensis*
