# Supplementary figures and images for: Co-infection of Sweet Orange with Severe and Mild Strains of Citrus tristeza virus Is Overwhelmingly Dominated by the Severe Strain on Both the Transcriptional and Biological Levels
Source: Front Plant Sci. 2017 Aug 31;8:1419. doi: 10.3389/fpls.2017.01419 (PMC5583216; doi:10.3389/fpls.2017.01419)

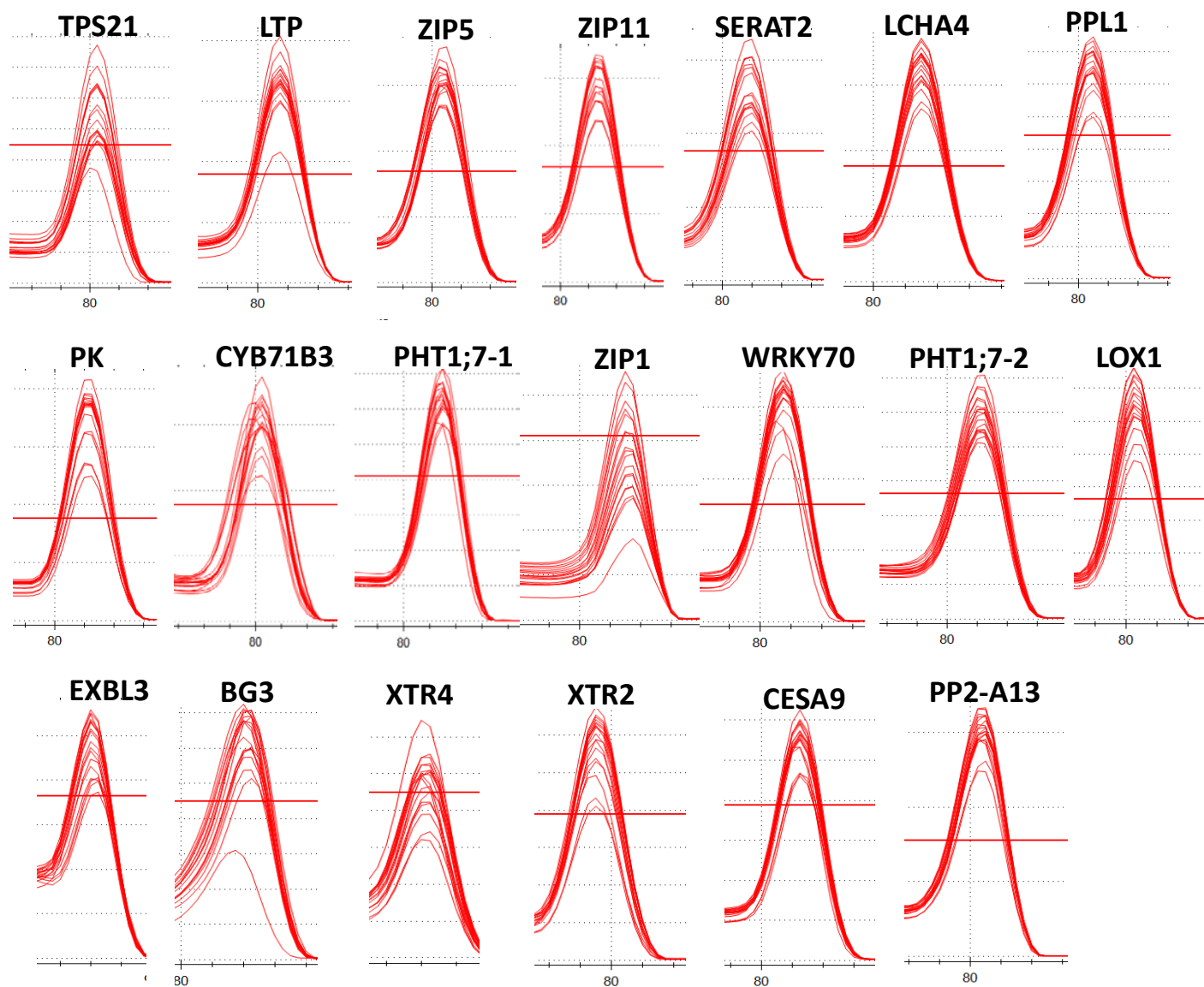

**Figure S3.** Melt curves of amplification products following RT-qPCR.

Supplement: Supplementary file 5 [file Image3.PDF]
